# Integrating administrative and clinical datasets to improve patient outcomes.

**DOI:** 10.23889/ijpds.v7i3.1892

**Published:** 2022-08-25

**Authors:** Jennifer Reid, Blayne Welk, Naheed Tahir, Amit Garg, Theresa Fitzgerald, Glen Kearns, Salimah Shariff

**Affiliations:** 1 ICES; 2 ICES, Western University; 3 London Health Sciences Centre, St. Joseph's Health Care London

Integrating electronic health records (EHR) with health administrative data offer opportunities for enhanced decision support, health systems evaluations and research for improved patient care. We describe the process of integrating EHR data from 12 hospitals in Southwestern Ontario, Canada and present a health systems evaluation enabled by this linkage.

With support and buy-in from the hospital Chief Executive Officers, a data sharing agreement (DSA) with ICES was executed, outlining the process to approve data transfer requests. Due to the complexity and volume of the EHR, complete data were not immediately transferred to ICES; instead, a project subcommittee comprising of physician leadership, local and regional privacy and risk officers, and ICES staff was assembled to review and approve project-specific data integration requests. An evaluation of the adoption of an electronic medication reconciliation system within the EHR on potentially inappropriate prescribing after hospital discharge was conducted using interrupted time-series analyses.

The data integration request process begins with confirmation of data availability and accuracy within the EHR, enabled by a dedicated health information analyst and institutional decision support teams. Once data feasibility is confirmed, project rationale is submitted to the project subcommittee approval. Following approval, the request undergoes privacy and legal assessment as per the DSA and a project-ready dataset is submitted for linkage.

An evaluation of the adoption of an electronic medication reconciliation system demonstrated an immediate and dramatic reduction in inappropriate medication prescribing and associated adverse events such as a fall or fracture among elderly patients discharged following acute inpatient stays.

Administrative data are valuable at assessing population-based health, but often lack key clinical information present in EHRs. Integrating both data sources offers a more comprehensive picture of patient care and allows for robust analyses. Rigorous investigations generate trusted evidence for decision makers to inform policy and quality improvements within the healthcare system.

